# RSA of the Symax hip stem

**DOI:** 10.1080/17453674.2020.1763042

**Published:** 2020-05-12

**Authors:** Hannu T Aro, Sanaz Nazari-Farsani

**Affiliations:** Department of Orthopaedics, Leiden University Medical Center, Leiden, The Netherlands

*Sir,*—We read with a great interest the recent article by Kruijntjens et al. (2020). The investigators performed a 2-year model-based radiostereometric analysis (RSA) of the uncemented Symax femoral stem. The article reported no previous RSA studies on the Symax stem but we have executed a randomized double-blind, placebo-controlled trial (RCT) on the primary stability of the Symax stem in 49 postmenopausal women (Aro et al. 2018). The trial included an extended RSA follow-up for 3–5 years and the follow-up of implant survival for 8–10 years. We want to highlight some methodological differences between the 2 studies which make comparisons of interest.

The results of the 2 studies complement each other. The stem design lead to early stabilization (within 4–12 weeks) in both studies. In our RCT, the stem migration did not respond to antiresorptive therapy. All stems, independent of the amount of initial migration, osseointegrated radiographically. No revision arthroplasty was performed. Due to the low rate of clinical failure (< 2% at 9 years), no meaningful analysis of an association between early stem migration and implant survival could be carried out. In this respect, the Symax stem resembled the outcome of 7 uncemented femoral stems recently analyzed in a meta-analysis (van der Voort et al. 2015).

Based on the literature, Kruijntjens et al. concluded that there is a substantial variability in the amount of initial subsidence between stem designs. However, the comparison of different studies is challenging because any variation of stem migration may reflect more the heterogeneity of the skeletal status of study populations than the characteristics of tested femoral stem designs. The study of Kruijntjens et al. included both sexes with a mean age of 59 (30–70) years. Osteoporosis was an exclusion criterion but the measurement of local and systemic BMDs was not reported. The mean subsidence of the stem was minimal (y-translation –1.0 mm, 95% confidence interval [CI] –3.4 to 1.4). The mean stem rotation (retroversion) was 2.4° (CI –2.2 to 7.0). In our RCT, only subjects with normal BMD had minimal stem subsidence (0.7 mm, CI 0.2–1.2) and rotation into retroversion (0.8°, CI 0.3–1.4). On the contrary, osteopenic and osteoporotic subjects exhibited more stem subsidence and rotation during the first 12 weeks after surgery. The primary stability of uncemented femoral stems is sensitive to adequate bone stock (Nazari-Farsani et al. 2020). It is reasonable that all RSA arthroplasty studies have a preoperative evaluation of local and systemic BMD, if a study protocol accepts recruitment of subjects (like postmenopausal women) at a known risk of low systemic BMD.

Our RCT was performed in collaboration with the implant manufacturer, facilitating the standard marker-based RSA. Kruijntjens et al. applied model-based RSA (Kaptein et al. 2006), with experts of this method as co-investigators. Indeed, model-based RSA is highly tempting for clinical trials. The results of marker-based and model-based RSA show high agreement (Nazari-Farsani et al. 2016). Looking at the model-based RSA data of Kruijntjens et al. there was a considerable variation (CI –1.2° to 1.8°) in double examinations of y-axis rotation. The stem rotation to retroversion also had variation (CI –2.2 to 7.0). It would be great to get a comment of the investigators. Was the variation due to a actual inter-individual difference of stem rotation or only due to inherent challenges of model-based RSA in measurement of stem rotation?

Finally, Kruijntjens et al. performed the baseline RSA prior to loading of the operated hip, during the first day after surgery. They suggested a similar approach for all RSA studies. The suggestion was made without performing a comparison of different imaging and rehabilitation protocols. The current recommendation (ISO 16087:2013) is to schedule baseline RSA measurements within 5 days postoperatively, preferably before weight-bearing. 2 published studies have performed the baseline RSA imaging when the patients still were anesthetized. Interestingly, these studies showed no migration of uncemented femoral stems (Ström et al. 2007) and acetabular cups (Wolf et al. 2010) during the first week after surgery. The RCT of Ström et al. even compared the effect of different weight-bearing regimen on stem migration. The degree of early weight-bearing (unrestricted versus partial weight-bearing) did not change the migration pattern. The initial stem migration does not seem to start with the first steps of postoperative weight-bearing but progressively only after 1 week. Thus, the current recommendation for timing the baseline RSA may be still appropriate.

**Hannu T Aro and Sanaz Nazari-Farsani**

Department of Orthopaedic Surgery and Traumatology, University of Turku and Turku University Hospital, Turku, Finland

*Email: hannu.aro@utu.f*i

*Sir,*—We would like to comment on the remarks of Aro and Sanaz Nazari-Farsani in their recent letter to the editor concerning our recent article in the *Acta Orthopaedica* ‘Early stabilization of the uncemented Symax hip stem in a 2-year RSA study’ (Kruijntjens et al. 2020).

We apologize to have missed the study of Aro et al. (2018) and not having mentioned it in our references. The explanation is simple, this study is hard to find when searching on RSA and Symax; there were no search results on PubMed, neither in Clinical Trials, simply because the brand name of the hip was not mentioned in the title nor in the abstract of the paper by Aro et al. Surprisingly however, relevant publications from our group regarding the Symax stem were not referred to in the article of Aro et al. (Kruijntjens et al. 2018; ten Broeke et al. 2012).

As the excellent paper by Aro et al. should have been discussed in our paper, we appreciate the opportunity now given to have this discussion.

Aro and Sanaz Nazari-Farsani state in their comment that the results of the 2 studies complement each other. In his letter he mentions that the stem design lead to early stabilization (within 4–12 weeks) in both studies. In our view there is however an important difference between our studies. We do not agree that Aro et al. showed stabilization within 4 weeks, being impossible when the first follow-up is at 3 months. The point of our article is that by performing early RSA (at day 1 postoperatively, and 1, 3, 6, 12, and 24 months postoperatively), we could detect a much earlier stabilization of the stem at 4 weeks. Our findings correspond better to the histomorphometric results seen in our earlier study, showing very early osseointegration which we attribute to the combination of the fit and fill characteristics of the stem geometry in combination with the highly bioactive properties of the Bonit-HA coating (ten Broeke et al. 2011).

From Aro’s study it can only be concluded that stabilization was reached at 3 months. Nevertheless, both the study by Aro and our study do not report early implant failures, despite different levels of migration before stabilization, implying that there is obviously a safe range for migration of this stem before it becomes at risk for early loosening. This was also confirmed by our international Symax study (Kruijntjens et al. 2018) as well as in the Danish register study by Edwards et al. (2018). All aforementioned studies show that early osseointegration and (good) survival of the stem is not negatively affected by osteopenic/osteoporotic bone conditions.

In contrast with the statement of Aro and Sanaz Nazari-Farsani we did not exclude patients with osteoporosis, but patients taking medication that may influence bone metabolism, in order not to introduce a potentially confounding factor. We are aware, from earlier literature, that antiresorptive medication as well as vitamin D with calcium influence bone mineral density (Venesmaa et al. 2001, Sköldenberg et al. 2011), but have probably no clinically relevant influence on migration of uncemented hip stems (Aro et al. 2018), let alone definitive stabilization. Therefore, differentiating between groups with or without osteoporosis would not contribute in answering our research question, which focused on the potential of this stem, with its particular geometry and coating characteristics, for early stabilization across different patient groups. Already from an earlier DEXA-study from our group on bone remodelling around the Symax stem (ten Broeke et al. 2012), it was clear that at 1-year follow-up all stems showed radiological evidence of stable bone ingrowth, independent of BMD at t_0_.

The conclusion of the article of Aro et al. also was that stem migration was not influenced by the use of zoledronic acid, although zoledronic acid treated patients maintained periprosthetic BMD better than the control group. This conclusion was also drawn in a study by Aro et al. using denosumab (Aro et al. 2019). In other words, one may question if there is a reason anyhow to differentiate between initial BMD for choosing a particular hip implant.

A further remark by Aro and Sanaz Nazari-Farsani was on the wide range of the stem (Y-axis) rotations both in the double examinations and in the follow-up measurements. It is well known and accepted that model-based RSA is less precise compared to marker-based RSA. Still model based RSA has other advantages and is suitable in clinical studies as was also demonstrated in a study from Aro’s group (Nazari-Farsani et al. 2016). Prins et al. (2008) demonstrated that the precision of the Elementary Geometrical Shape (EGS) model-based approach (used in our study) was found to be acceptable for use in a clinical study. It is important to realize that by increasing the number of patients, the lower precision of model-based RSA can be mended, as the accuracy (the bias) is as low as for marker-based RSA. During the settling of the stem in the initial month postoperatively, the stem also rotates into retroversion. The difference in CI between the double examinations and clinical data show that the range in rotation in our study, is mainly the result of the variation in actual rotation between patients, in combination with the variation introduced by the slightly less precision caused by using a model-based RSA approach. But most important, as stated in our paper, the initial level of migration of the uncemented stem is less relevant compared to the stabilisation of the stem in the period after initial settling to predict long term survival of the stem.

Finally, we completely agree that the current recommendation for timing the baseline RSA is still appropriate, as our recommendation is exactly the same as stated in the ISO standard (ISO 16087:2013) on RSA. Especially for uncemented hip stems, we do recommend to add an extra early follow-up moment at 1 month in order to have more exact data on the early post-operative migration patterns.

**Dennis S M G Kruijntjens, Lennard Koster, Bart L Kaptein, Jacobus J C Arts, and René H M ten Broeke**

Departments of Orthopaedic Surgery, Research School Caphri, Maastricht University Medical Centre, Maastricht;

Department of Orthopaedic Surgery, RSAcore, Leiden University Medical Centre, Leiden, the Netherlands

*Email:* d.kruijntjens@gmail.com
